# Relaxometric learning: a pattern recognition method for *T*_2_ relaxation curves based on machine learning supported by an analytical framework

**DOI:** 10.1186/s13065-020-00731-0

**Published:** 2021-02-20

**Authors:** Yasuhiro Date, Feifei Wei, Yuuri Tsuboi, Kengo Ito, Kenji Sakata, Jun Kikuchi

**Affiliations:** 1 RIKEN Center for Sustainable Resource Science, 1-7-22 Suehiro-cho, Tsurumi-ku, Yokohama, Kanagawa 230-0045 Japan; 2 Graduate School of Medical Life Science, Yokohama City University, 1-7-29 Suehiro-cho, Tsurumi-ku, Yokohama, Kanagawa 230-0045 Japan; 3 Graduate School of Bioagricultural Sciences, Nagoya University, 1 Furo-cho, Chikusa-ku, Nagoya, Aichi 464-8601 Japan

**Keywords:** Machine learning, Nuclear magnetic resonance, Relaxometry, Support vector machine, Geographical origin determination

## Abstract

Nuclear magnetic resonance (NMR)-based relaxometry is widely used in various fields of research because of its advantages such as simple sample preparation, easy handling, and relatively low cost compared with metabolomics approaches. However, there have been no reports on the application of the *T*_2_ relaxation curves in metabolomics studies involving the evaluation of metabolic mixtures, such as geographical origin determination and feature extraction by pattern recognition and data mining. In this study, we describe a data mining method for relaxometric data (i.e., relaxometric learning). This method is based on a machine learning algorithm supported by the analytical framework optimized for the relaxation curve analyses. In the analytical framework, we incorporated a variable optimization approach and bootstrap resampling-based matrixing to enhance the classification performance and balance the sample size between groups, respectively. The relaxometric learning enabled the extraction of features related to the physical properties of fish muscle and the determination of the geographical origin of the fish by improving the classification performance. Our results suggest that relaxometric learning is a powerful and versatile alternative to conventional metabolomics approaches for evaluating fleshiness of chemical mixtures in food and for other biological and chemical research requiring a nondestructive, cost-effective, and time-saving method. 
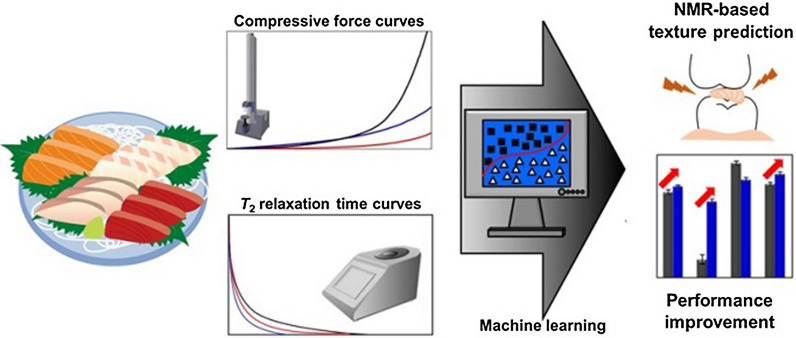

## Introduction

Nuclear magnetic resonance (NMR) spectroscopy is one of the most versatile tools for chemical analysis in the fields of chemistry and biology [1]. NMR can be used to evaluate complex chemical and biological mixtures (e.g., those used in metabolomics studies), which characterize the metabolic profiles of a large number of samples derived from biological and environmental systems [2]. NMR-based metabolomics is used to determine geographical provenance, solve problems related to food fraud, and certify a “terroir” in food chemistry [3]. However, conventional NMR-based metabolomics approaches require relatively high-field NMR instruments to obtain high-resolution NMR spectra. Therefore, although recent advances in benchtop NMR instruments have been accompanied by increasing employment of low-field and benchtop NMR spectroscopy in NMR-based metabolomics studies, the practical utilization and on-site application of NMR-based metabolomics are limited [4].

As an alternative to metabolomics approach, NMR-based relaxometry has several advantages, including compatibility with low-field and compact NMR instruments, simple sample preparation, easy handling, and relatively low cost compared with metabolomics approach [5]. Therefore, NMR-based relaxometry is widely used in various research fields and industries such as the food industry, polymer industry, pharmaceutical industry, and geology. However, the application of the *T*_2_ relaxation curves obtained by relaxometric measurements to biological studies has not yet been reported in terms of the characterization and evaluation of chemical mixtures to determine the geographical origin and achieve feature extraction by pattern recognition and data mining.

The *T*_2_ relaxation curves obtained by relaxometric measurements appear to be relatively simple compared with the ^1^H NMR spectra obtained in conventional metabolomics studies. However, the *T*_2_ relaxation curves derived from complex chemical mixtures (e.g., fish and vegetables in the field of food chemistry) are likely to show slightly different profiles for each sample because of complex bound water states and interactions that are affected by the higher-order structure of macromolecules, although discerning such small differences by eye is usually difficult (Additional file 1: Fig. S1). With this in mind, we speculated that data mining methods such as multivariate analyses and machine learning (ML) could be applied to discover valuable information buried in *T*_2_ relaxation curves.

Multivariate analysis methods such as principal component analysis, partial least squares (PLS), and soft independent modeling of class analogy [6] have been used to analyze relaxometric data [7–9]; however, the use of ML methods for such analyses has not been reported. Compared with multivariate analyses, ML methods are known to be superior for metabolomics studies in some situations [10], and several useful ML-incorporated analytical tools have been reported (e.g., MetaboAnalyst [11] and KODAMA [12]). In a previous work, we successfully developed several ML-based analytical approaches, namely, a prediction method for metabolic mixture signals [13], deep neural network (DNN)–mean decrease accuracy [14] and ensemble DNN [15] methods, variable selection for regional feature extraction [16], evaluation of surface water [17], impact estimation of food intake on mice [18], and evaluation of daily dietary intakes of humans [19] in metabolomics studies. Thus, we considered that an analytical method for *T*_2_ relaxation curves combined with ML might be a helpful tool for metabolomics studies and could be used to discriminate between geographical origins and extract features pertaining to sample attributes.

In this study, we describe a technique called relaxometric learning, which is a pattern recognition method for *T*_2_ relaxation curves that can be used as a simple and cost-effective tool for the data mining of complex chemical and biological mixtures (e.g., fish samples in food chemistry) as an alternative to conventional metabolomics approaches. To develop the relaxometric learning, an analytical framework optimized for data mining of *T*_2_ relaxation curves was required. Therefore, we also developed an analytical framework for eliciting the improved classification performance of ML algorithms to enhance the performance of relaxometric learning. In the analytical framework, we incorporated two methods into the ML process: a variable optimization approach to enhance the classification performance and bootstrap resampling-based matrixing to balance the sample size between groups. As a model case, we selected the support vector machine (SVM) [20] approach for analyzing the *T*_2_ relaxation curves obtained by NMR-based relaxometric measurements because SVM has been reported to be useful for analyses with a relatively small number of samples [21]. The classification performance of relaxometric learning was evaluated on the basis of the physical properties (hardness and tenderness) of fish muscles determined by the hierarchical cluster analysis (HCA) of compressive force data measured by an autograph machine in a data-driven manner. The applicability of the analytical framework to other ML methods was also evaluated.

## Materials and methods

### Sample collection

A total of 233 fish samples belonging to 34 families were collected from April 2012 to November 2018 at multiple coastal sites in Japan (Additional file 1: Table S1). There is no specific permission required for all of the sampling points as they are all public places. The animal experiments were performed in accordance with protocols approved by the Institutional Committee of Animal Experiment of RIKEN and adhered to the guidelines in the Institutional Regulation for Animal Experiments and Fundamental Guidelines for Proper Conduct of Animal Experiment and Related Activities in Academic Research Institutions under the jurisdiction of the Ministry of Education, Culture, Sports, Science and Technology, Japan. Among these samples, 233 and 209 samples (the different sample number was due to insufficient volume of fish muscles) were used for compressive force measurements by autograph and NMR measurements to obtain *T*_2_ relaxation curves, respectively.

### Compressive force measurements by autograph

Since the relaxation properties and water contents in fish muscle could be varied according to position differences [22], fish muscle above the anal fin was picked to avoid the impact of position differences and cut into slices (5 mm thick and 10 mm wide) (Additional file 1: Fig. S2A). Stress testing (n = 5 per sample) was performed using a multipurpose stretching tester comprising an autograph (EZ-L, Shimadzu Co. Ltd., Kyoto, Japan) with a wedge-shaped cutter bit (Additional file 1: Fig. S2B). The parameters were controlled using TRAPEZIUM2 (ver. 2.36, Shimadzu), which is the manufacturer-supplied software. The loading rate was 2 mm min^−1^, the total distance was 5 mm (Additional file 1: Figs. S2C and S2D), and the force–time curves were recorded (Additional file 1: Fig. S2E). A total of 1165 curves were obtained in the measurements.

The force–time curves for fish muscle were transformed into force–distance curves, with the zero point determined by the contact between the sample and the cutter bit. The portions of the curves between 0 and 3.63 mm were selected (34 curve data were omitted in this process due to the insufficient length (thickness)), and exponential fitting was performed in Microsoft Excel by using the following equation:

1 $$y = ae^{bx} ,$$

where *x* represents the distance from the starting (zero) point, *y* represents the compressive force, and *a* and *b* represent the fitting coefficients.

A total of 1131 sets of compressive force data were preprocessed, and coefficients *a* and *b* were calculated by approximating the exponential function. Coefficients *a* and *b* were further analyzed using a data-driven HCA approach, which indicated that each sample could be categorized into two groups on the basis of the physical properties (i.e., hardness and tenderness) of the fish muscle (Additional file 1: Fig. S3). The fish belonging to group A had relatively hard muscles compared with those in group B.

### NMR measurements to obtain *T*_2_ relaxation curves

A piece of fish muscle (n = 3 per sample) was loaded into a 5 mm NMR tube inserting a 2 mm NMR tube filled with 99.9% D_2_O solvent for locking. *T*_2_ relaxation curves were recorded at 298 K on a high-resolution NMR spectrometer (AvanceIII HD-500, Bruker, Rheinstetten, Germany) equipped with a ^1^H inverse probe with triple resonance by using the Bruker standard pulse program “cpmg_T2_1d” (Carr–Purcell–Meiboom–Gill sequence) with 1 scan, 512 data points, and 1.024 s acquisition time. The obtained raw data were normalized by unit variance and used for further analyses.

### Data analysis

**Fig. 1:**
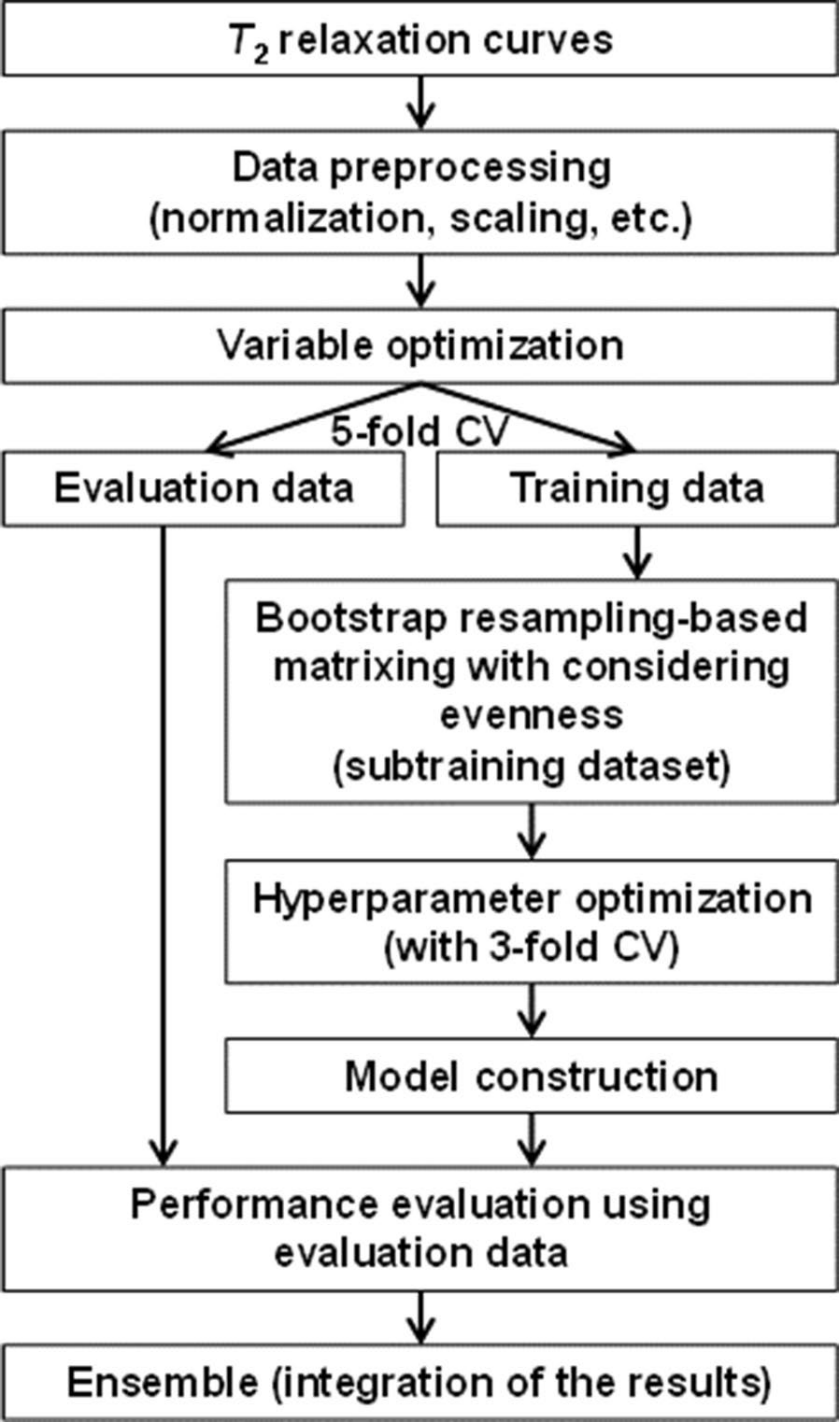
Analytical flow of relaxation curve data during relaxometric learning. Compressive force data analyzed by HCA were used as explanatory variables for the analyses of *T*_2_ relaxation curves in a data-driven manner. The relaxation curves were preprocessed and analyzed by ML incorporating an analytical framework including a variable optimization approach and bootstrap resampling-based matrixing. The results obtained for each classifier were finally integrated, similar to that in ensemble learning

HCA was performed using Ward’s method and was implemented using the “hclust” function in the R software package (version 3.2.2) [23]. SVM, random forest (RF) [24], and PLS were performed using the e1071 [14], randomForest [14], and pls [25] libraries, respectively, with repeated (10 times) double cross-validation (CV) [2] in R. In the double CV, threefold CV and fivefold CV were used for the hyperparameter optimization and performance evaluation of the constructed models, respectively (Fig. 1). The SVM hyperparameters were evaluated at the ranges of 0.0001 to 1 for gamma and 1 to 1000 for cost, and the most frequently determined values in the hyperparameter optimization process of the double CV were 0.03 and 5 for gamma and cost, respectively. Receiver operating characteristic (ROC) curves and the area under the curve (AUC) were calculated using the ROCR library [26].

## Results and discussion

### Classification performance of SVM for ***T***_2_ relaxation curve data

The applicability of ML algorithms to the data mining of *T*_2_ relaxation curves was firstly evaluated because such analyses have not been reported in the literature, and several ML approaches have shown relatively good performance in classification problems compared with multivariate analyses [14]. Conventional SVM classification, which is a typical ML method, was performed using category information from two groups of compressive force data as explanatory variables for the *T*_*2*_ relaxation curve data (a total of 627 curves) of various fish muscle samples collected from multiple coastal sites in Japan. Unfortunately, the classification performance was worse than expected; the AUC, accuracy, correctly classified rate for group A (CCR-A), and correctly classified rate for group B (CCR-B) were 0.780, 0.748, 0.475, and 0.865, respectively (Additional file 1: Table S2).

### Variable optimization to enhance the classification performance

To improve the SVM classification performance, we used a variable optimization approach to enhance the quality of information obtained from simple *T*_2_ relaxation curve data. The variable optimization approach employed was a search method that determined the best length of raw curve to improve the classification performance via the reduction of variables from long- to short-relaxation-time components in sequential order. This variable reduction idea is based on the elimination of “noise” variables, which possibly arise from background noise and/or from free water, i.e., relatively long *T*_2_ relaxation time components in the relaxation curve are barely related to the characteristic features of samples and were suspected of interfering with the accuracy of the SVM learning step.

In this study, the variables were gradually reduced at the rate of 10% from all variables (the number of variables was 256) to 10% usage rate (25 variables) by omitting the relatively long *T*_2_ relaxation time components. For instance, the dataset at 90% usage rate included 230 variables (from 0 to 0.92 s of *T*_2_ relaxation time components) and more than 0.92 s of the components was removed. The each dataset generated by the variable reductions was applied to performance evaluation by SVM classification. Based on the classification performance, the optimized number of variables was determined.

**Fig. 2:**
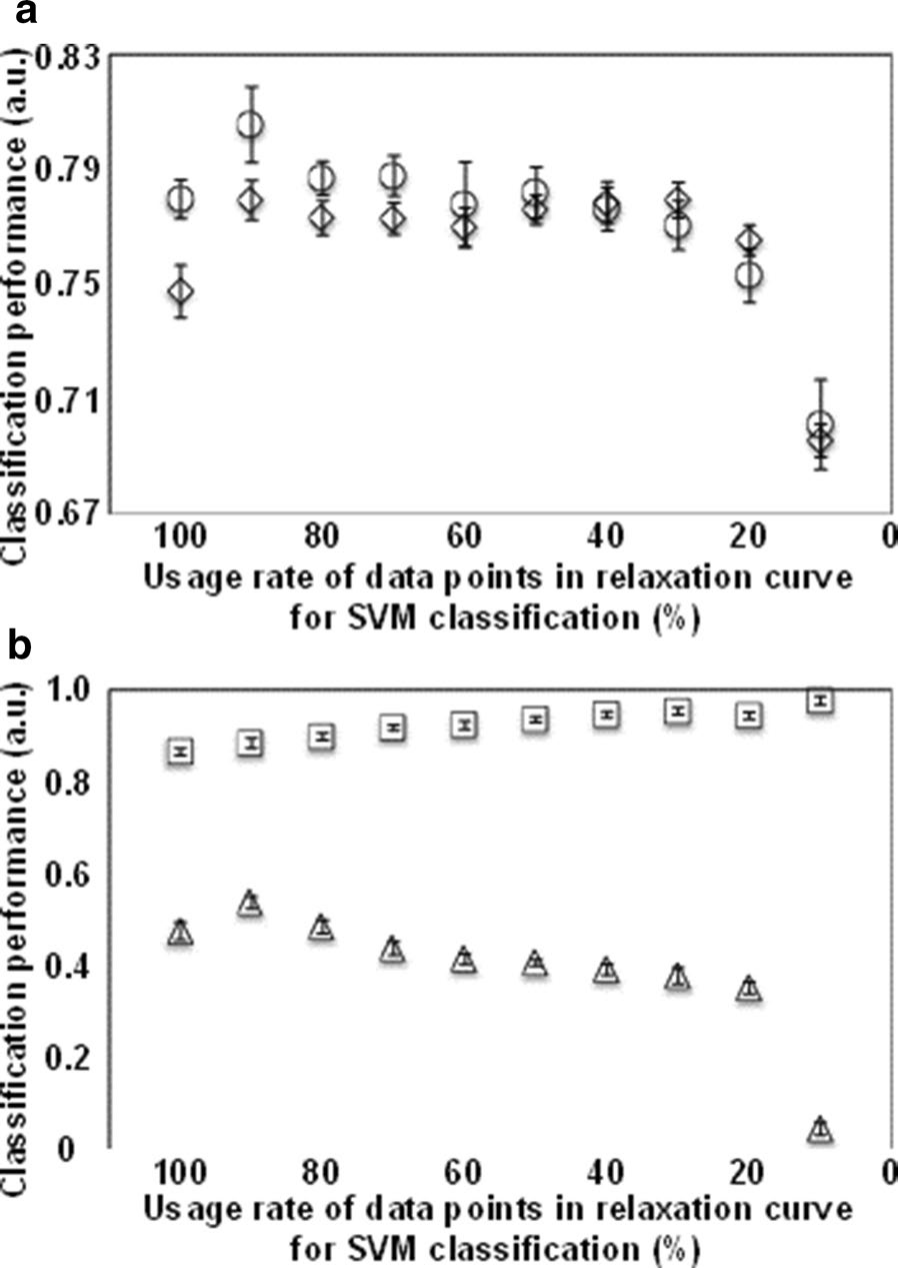
Performance evaluation of the variable optimization approach based on conventional SVM classifications. **a** AUC (circles) and accuracy (diamonds). **b** CCR-A (triangles) and CCR-B (squares)

The incorporation of the variable optimization approach into the SVM classification method improved the classification performance for the two groups (Fig. 2). The best SVM classification performance was obtained with a 10% reduction (90% usage rate) of data points (variables) from the *T*_2_ relaxation curve, with AUC and accuracy values of 0.806 and 0.779. Although the value of CCR-A was also improved from 0.475 to 0.538, the relatively low value still required improvements to obtain satisfactory classification performance.

### Bootstrap resampling to balance the sample size between groups

**Fig. 3:**
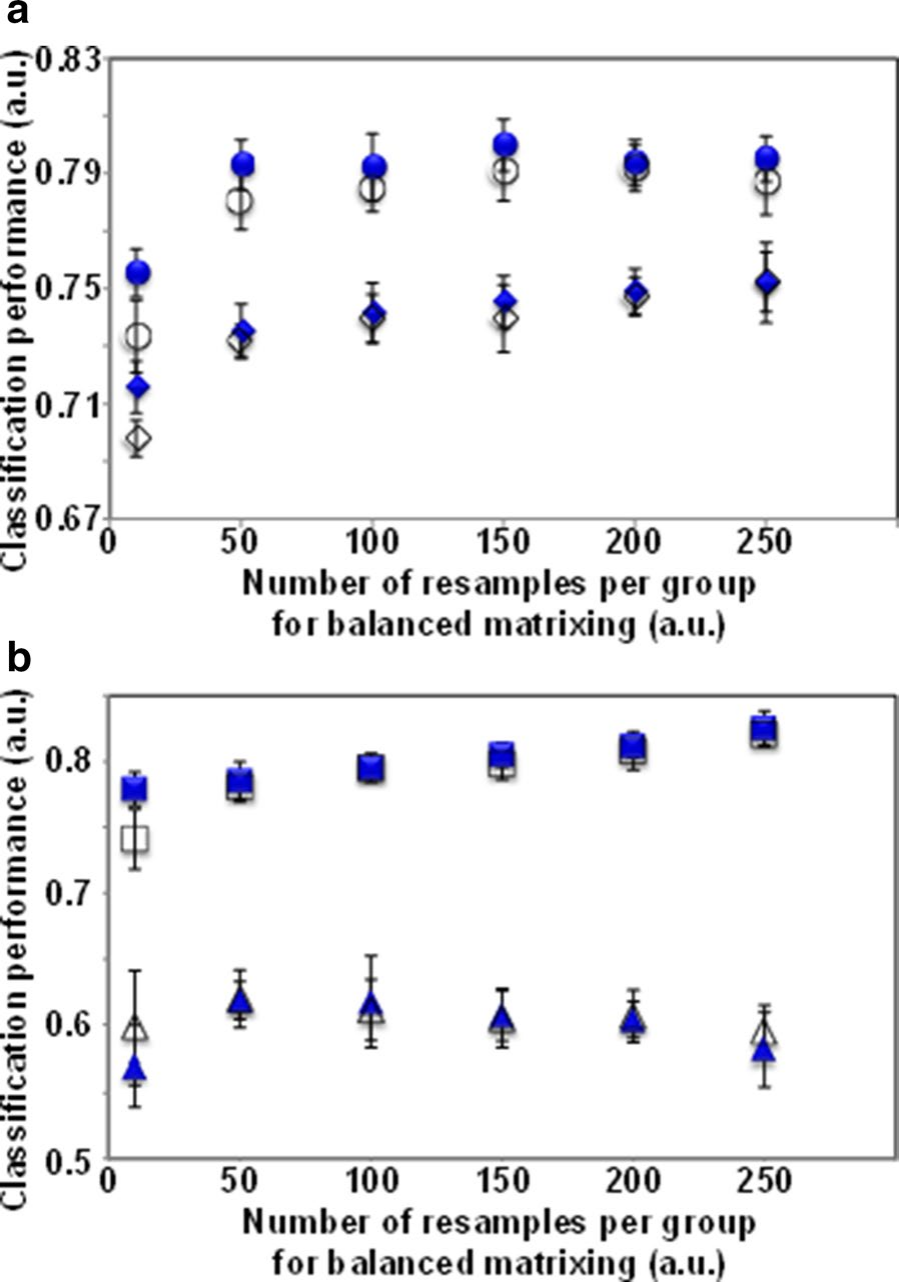
Performance evaluation of the bootstrap resampling-based matrixing approach based on conventional SVM classifications. The performance of the approach was evaluated with the number of datasets generated by the bootstrap resampling set at 100 (closed blue symbols) and 10 (open symbols) matrices. **a** AUC (circles) and accuracy (diamonds). **b** CCR-A (triangles) and CCR-B (squares)

**Fig. 4:**
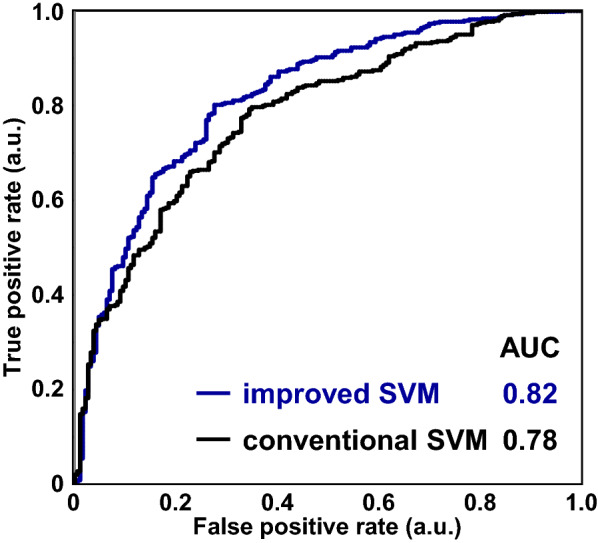
ROC curves and the corresponding AUC values obtained using conventional SVM (black) and SVM-based relaxometric learning (blue)

The classification performances of conventional SVM with and without the above variable optimization approach were profoundly affected by the biased sample size between groups in the data matrix of the *T*_2_ relaxation curve. To circumvent this difficulty, we focused on a bootstrap resampling-based matrixing that constructs a matrix (subtraining dataset) by considering the evenness of the sample size between groups for the model construction of ML classifications (Fig. 1). In this approach, the hyperparameters (i.e., the number of resamples for balanced matrixing and the number of datasets generated by bootstrap resampling) were evaluated (Fig. 3). When the number of generated datasets was set to 100, a slightly better performance was obtained in terms of AUC and accuracy compared with only 10 generated datasets, whereas different resample sizes yielded almost the same AUC and accuracy values, except for resample sizes below 50 per group (a total of 100 samples per dataset). Furthermore, the CCR-A slightly decreased with increasing resamples. Therefore, we performed variable optimization combined with bootstrap resampling-based matrixing by using 100 generated datasets with 150 resamples (a total of 300 samples) per dataset (Additional file 1: Fig. S4). At the 90% level of variable usage rates for *T*_2_ relaxation curves exhibiting the best SVM classification performance, the classification performance of the constructed analytical framework was significantly improved compared with conventional SVM in terms of the ROC curve and the AUC value (Fig. 4). The AUC, accuracy, and CCR-A values significantly increased from 0.780, 0.748, and 0.475 to 0.820, 0.771, and 0.710, respectively (Additional file 1: Fig. S5). In addition, robustness evaluation of the developed method for fluctuation of each variable was performed using datasets generated by random resampling based on permutation for a variable (Additional file 1: Fig. S6). The fluctuation of each variable had relatively little effect on the SVM classification performance using the analytical framework developed in this study, indicating that the developed method enables to construct robust models for variable fluctuation. Therefore, the analytical framework described here, namely, the incorporation of the variable optimization approach and the bootstrap resampling-based matrixing into the ML calculations, resulted in improved classification performance in the data mining of *T*_2_ relaxation curve data and enhanced the robustness when using unbalanced datasets. Furthermore, the relaxometric learning method developed here enabled the extraction of features related to the physical properties (hardness and tenderness) of fish muscle.

### Applicability of the analytical framework to other machine learning methods

**Fig. 5:**
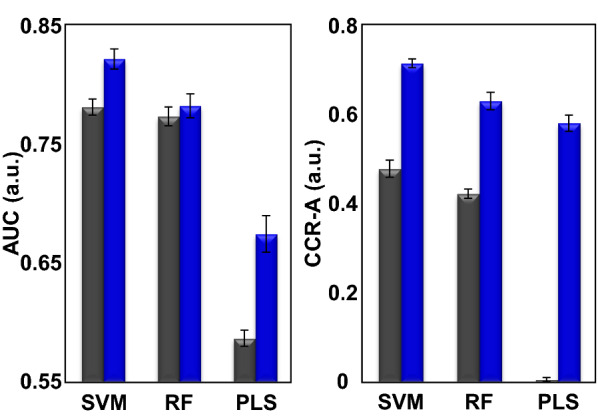
Comparison of classification performance between relaxometric learning (blue bars) and conventional MLs (black bars). The SVM, RF, and PLS algorithms were employed. The standard deviations are displayed as error bars. Left panel, AUC; right panel, CCR-A

The analytical framework developed in this study is optimized for data mining of *T*_2_ relaxation curve data. Thus, we considered that not only SVM but also other ML algorithms and multivariate analyses may be useful for the data mining of *T*_2_ relaxation curve data. To test this hypothesis, RF and PLS were used as alternatives to SVM for data mining based on the developed analytical framework. The classification performance of RF improved slightly in terms of the ROC curve and the AUC value, and the CCR-A values were significantly improved by the incorporation of our analytical framework (Fig. 5 and Additional file 1: S7). On the other hand, the classification performance of PLS improved drastically in terms of the values of both AUC and CCR-A (Fig. 5 and Additional file 1: S7). These results suggest that our analytical framework is applicable to various ML algorithms and multivariate analyses to enhance classification performance, but the extent of improvement is method dependent. Therefore, the relaxometric learning approach developed in this study should find use as a versatile and useful method for the analysis of *T*_2_ relaxation curve data.

### Applicability of relaxometric learning to the determination of geographical origin

NMR-based metabolomics approaches are capable of determining the geographical origins of food products such as fish [14, 16, 27–29], beef [30], durum wheat [31], white rice [32], apple [33], cabbage [34], honey [35], coffee beans [36], and wine [37]. Therefore, relaxometric learning was also considered applicable for such analyses. In addition, the quality of biological tissue (such as water content and water activity) in different environment is varied according to the geographical origins [38]. Here, we proposed that the difference in the water conditions could be detected by pattern recognition of *T*_2_ relaxation curves using NMR but not high performance liquid chromatography or mass spectrometry methods. Then, we performed experiments to evaluate the applicability of relaxometric learning to discriminate between geographical differences (i.e., to extract features in terms of the habitats of *Girella punctata* belonged to Kyphosidae fish living in Tokyo Bay and Sagami Bay) based on *T*_2_ relaxation curves (Additional file 1: Fig. S8). The SVM-based relaxometric learning method exhibited relatively good performance in the geographical origin discrimination of fish compared with conventional SVM, thus leading to a significant increase in AUC value from 0.886 to 0.936 (Additional file 1: Fig. S8). These results suggest that relaxometric learning is applicable as a method for determining geographical provenance, solving problems related to food fraud, and certifying the “terroir” of food, similar to the case for conventional metabolomics approaches.

Compact benchtop or portable NMR spectrometers are low-cost alternatives to conventional high-field and high-resolution spectrometers. Benchtop low-field NMR spectrometers can theoretically obtain *T*_2_ relaxation curves with a similar quality to those obtained with high-resolution NMR spectrometers, such as the one used in this study; therefore, similar classification accuracies can be expected. Relaxometric learning using benchtop and portable NMR spectrometers even without using D_2_O might also find applications in on-site quality control and fleshiness management, optimization of production processes, and improvement of product quality not only in food but also in various industrial fields such as polymers, cosmetics, fabrics, pharmaceuticals, and healthcare. Relaxometric learning is expected to be a versatile and powerful approach for the characterization and evaluation of industrial products and as an option for biological and chemical research that requires a nondestructive, cost-effective, and time-saving method.

## Conclusions

This study focused on the development of a new relaxometric learning method based on the pattern recognition of *T*_2_ relaxation curves. The method is supported by an analytical framework incorporating a variable optimization approach and bootstrap resampling-based matrixing to enhance the classification performance of the ML algorithms employed. The developed relaxometric learning approach enabled the extraction of several features of fish muscles, such as their physical properties and geographical origin, from *T*_2_ relaxation data. Relaxometric learning was also implemented with not only SVM but also other ML and multivariate methods for the analysis of *T*_2_ relaxation data. The SVM-based relaxometric learning method was superior to the conventional SVM method, thus indicating that the analytical framework constructed in this study enables better classification performance when ML algorithms are applied to relaxation curve data. The relaxometric learning approach is a versatile, cost-effective, and time-saving tool for characterizing physical properties, such as the fleshiness of fish muscles; for evaluating product qualities, such as geographical origin; and for food authentication in biological and chemical samples.

## Supplementary Information

**Additional file 1: Table S1.** List of fish samples used in this study, **Table S2.** Classification performance of conventional SVM, **Figure S1.** Representative *T*_2_ relaxation curves for the various fish samples used in this study, **Figure S2.** Analytical procedure involving compressive force measurements by autograph, **Figure S3.** Categorization of compressive force data based on a data-driven approach, **Figure S4.** Performance evaluation of variable optimization approach with bootstrap resampling-based matrixing, **Figure S5.** Classification performance of SVM-based relaxometric learning, **Figure S6.** Robustness evaluation of the relaxometric learning for fluctuation of each variable. **Figure S7.** ROC curves and the corresponding AUC values for each method, **Figure S8.** Classification performance of SVM-based relaxometric learning in determining the geographical differences between Kyphosidae taken from Tokyo Bay and Sagami Bay.

## Data Availability

The complete database is accessible in the website http://dmar.riken.jp/NMRinformatics/.

## Abbreviations

AUC: Area under the curve
CCR-A: Correctly classified rate for group A
CCR-B: Correctly classified rate for group B
CV: Cross-validation
DNN: Deep neural network
HCA: Hierarchical cluster analysis
NMR: Nuclear magnetic resonance
ML: Machine learning
PLS: Partial least squares
RF: Random forest
ROC: Receiver operating characteristic
SVM: Support vector machine
