# Supplementary material for: Relaxometric learning: a pattern recognition method for T2 relaxation curves based on machine learning supported by an analytical framework
Source: BMC Chem. 2021 Feb 20;15(1):13. doi: 10.1186/s13065-020-00731-0 (PMC7897374; doi:10.1186/s13065-020-00731-0)
Supplement: Supplementary file 1 — Additional file 1: Table S1. List of fish samples used in this study, Table S2. Classification performance of conventional SVM, Figure S1. Representative T2 relaxation curves for the various fish samples used in this study, Figure S2. Analytical procedure involving compressive force measurements by autograph, Figure S3. Categorization of compressive force data based on a data-driven approach, Figure S4. Performance evaluation of variable optimization approach with bootstrap resampling-based matrixing, Figure S5. Classification performance of SVM-based relaxometric learning, Figure S6. Robustness evaluation of the relaxometric learning for fluctuation of each variable. Figure S7. ROC curves and the corresponding AUC values for each method, Figure S8. Classification performance of SVM-based relaxometric learning in determining the geographical differences between Kyphosidae taken from Tokyo Bay and Sagami Bay. [file 13065_2020_731_MOESM1_ESM.docx]

**Supplementary Information**

**Relaxometric learning: A pattern recognition method for *T_2_* relaxation curves based on machine learning supported by an analytical framework**

Yasuhiro Date^1,2^, Feifei Wei^1^, Yuuri Tsuboi^1^, Kengo Ito^1^, Kenji Sakata^1^ and Jun Kikuchi^1,2,3,^*

^1^RIKEN Center for Sustainable Resource Science, 1-7-22 Suehiro-cho, Tsurumi-ku, Yokohama, Kanagawa 230-0045, Japan.

^2^Graduate School of Medical Life Science, Yokohama City University, 1-7-29 Suehiro-cho, Tsurumi-ku, Yokohama, Kanagawa 230-0045, Japan.

^3^Graduate School of Bioagricultural Sciences, Nagoya University, 1 Furo-cho, Chikusa-ku, Nagoya, Aichi 464-8601, Japan.

**Contents:**

**Table S1. List of fish samples used in this study.**

**Table S2. Classification performance of conventional SVM.**

**Figure S1. Representative *T_2_* relaxation curves for the various fish samples used in this study.**

**Figure S2. Analytical procedure involving compressive force measurements by autograph.**

**Figure S3. Categorization of compressive force data based on a data-driven approach.**

**Figure S4. Performance evaluation of variable optimization approach with bootstrap resampling-based matrixing.**

**Figure S5. Classification performance of SVM-based relaxometric learning.**

**Figure S6. Robustness evaluation of the relaxometric learning for fluctuation of each variable.**

**Figure S7. ROC curves and the corresponding AUC values for each method.**

**Figure S8. Classification performance of SVM-based relaxometric learning in determining the geographical differences between Kyphosidae taken from Tokyo Bay and Sagami Bay.**

**Table S1.** **List of fish samples used in this study.**

| **Group** | **Family name** | **Sampling date** | **Sampling site** | **Body length (mm)** | **Weight (g)** |
| --- | --- | --- | --- | --- | --- |
| A | Balistidae | 25-Jun-16 | Ishigaki Island | 156 | 171 |
| A | Balistidae | 18-Jun-17 | Okinawa Islands | 180 | 294 |
| A | Balistidae | 18-Jun-17 | Okinawa Islands | 205 | 308 |
| A | Balistidae | 22-Oct-18 | Okinawa Islands | 215 | 331 |
| A | Carangidae | 02-Dec-17 | Tokyo bay | 410 | 1142 |
| A | Carangidae | 09-Dec-17 | Tokyo bay | 410 | 956 |
| A | Carangidae | 09-Dec-17 | Tokyo bay | 365 | 915 |
| A | Carangidae | 09-Dec-17 | Tokyo bay | 410 | 1214 |
| A | Carangidae | 09-Dec-17 | Tokyo bay | 410 | 1031 |
| A | Carangidae | 09-Dec-17 | Tokyo bay | 400 | 1086 |
| A | Carangidae | 09-Dec-17 | Tokyo bay | 390 | 837 |
| A | Carangidae | 22-Oct-18 | Okinawa Islands | 302 | 677 |
| A | Carangidae | 18-Nov-18 | Sagami bay | 250 | 271 |
| A | Carangidae | 22-Sep-18 | Sagami bay | 350 | 759 |
| A | Centrarchidae | 23-Jun-18 | Tamagawa river | 232 | 398 |
| A | Chaetodontidae | 31-Oct-16 | Okinawa Islands | 123 | 123 |
| A | Congridae | 30-Sep-17 | Tomakomai port | -^*1^ | 177 |
| A | Congridae | 30-Sep-17 | Tomakomai port | -^*1^ | 142 |
| A | Cyprinidae | 01-Oct-17 | Lake Shikotsu | 217 | 162 |
| A | Cyprinidae | 08-Oct-17 | Tonegawa river | 252 | 561 |
| A | Cyprinidae | 08-Oct-17 | Tonegawa river | 213 | 339 |
| A | Cyprinidae | 08-Oct-17 | Tonegawa river | 205 | 267 |
| A | Cyprinidae | 28-Oct-17 | Lake Kasumigaura | 155 | 107 |
| A | Cyprinidae | 28-Oct-17 | Lake Kasumigaura | 267 | 587 |
| A | Cyprinidae | 08-Oct-17 | Tonegawa river | 210 | 178 |
| A | Cyprinidae | 08-Jul-18 | Tonegawa river | 768 | 8520 |
| A | Cyprinidae | 15-Jul-18 | Tonegawa river | 785 | 11100 |
| A | Cyprinidae | 04-Aug-18 | Tonegawa river | 750 | 8700 |
| A | Holocentridae | 08-Apr-17 | Okinawa Islands | 192 | 224 |
| A | Ictaluridae | 08-Oct-17 | Tonegawa river | 252 | 269 |
| A | Ictaluridae | 28-Oct-17 | Lake Kasumigaura | 420 | 1443 |
| A | Ictaluridae | 28-Oct-17 | Lake Kasumigaura | 330 | 635 |
| A | Lateolabracidae | 02-Dec-17 | Tokyo bay | 328 | 474 |
| A | Lethrinidae | 28-Jan-17 | Ishigaki Island | 209 | 222 |
| A | Lethrinidae | 22-Oct-18 | Okinawa Islands | 265 | 507 |
| A | Lethrinidae | 22-Oct-18 | Okinawa Islands | 265 | 506 |
| A | Lethrinidae | 22-Oct-18 | Okinawa Islands | 265 | 497 |
| A | Lethrinidae | 22-Oct-18 | Okinawa Islands | 260 | 480 |
| A | Lethrinidae | 22-Oct-18 | Okinawa Islands | 255 | 453 |
| A | Lethrinidae | 21-Oct-18 | Okinawa Islands | 221 | 300 |
| A | Monacanthidae | 25-Oct-18 | Shirahama | 210 | 401 |
| A | Mullidae | 28-Jan-17 | Ishigaki Island | 185 | 137 |
| A | Mullidae | 18-Jun-17 | Okinawa Islands | 219 | 333 |
| A | Mullidae | 20-Oct-18 | Okinawa Islands | 197 | 214 |
| A | Mullidae | 20-Oct-18 | Okinawa Islands | 184 | 184 |
| A | Paralichthyidae | 26-Aug-18 | Sagami bay | 385 | 974 |
| A | Pinguipedidae | 01-Nov-16 | Okinawa Islands | 105 | 22 |
| A | Platycephalidae | 26-Aug-18 | Sagami bay | 440 | 764 |
| A | Pleuronectidae | 30-Sep-17 | Tomakomai port | 165 | 109 |
| A | Pleuronectidae | 12-Nov-16 | Tokyo bay | 252 | 376 |
| A | Pleuronectidae | 12-Nov-16 | Tokyo bay | 250 | 557 |
| A | Pleuronectidae | 12-Nov-16 | Tokyo bay | 193 | 158 |
| A | Pleuronectidae | 12-Nov-16 | Tokyo bay | 165 | 125 |
| A | Scombridae | 13-Aug-17 | Sagami bay | 297 | 478 |
| A | Scombridae | 19-Aug-18 | Sagami bay | 250 | 254 |
| A | Scombridae | 19-Aug-18 | Sagami bay | 239 | 182 |
| A | Scombridae | 19-Aug-18 | Sagami bay | 260 | 253 |
| A | Scombridae | 19-Aug-18 | Sagami bay | 249 | 216 |
| A | Scombridae | 02-Sep-18 | Tokyo bay | 215 | 152 |
| A | Scombridae | 29-Sep-18 | Sagami bay | 371 | 1089 |
| A | Scombridae | 29-Sep-18 | Sagami bay | 384 | 1237 |
| A | Scombridae | 29-Sep-18 | Sagami bay | 375 | 1241 |
| A | Scombridae | 08-Oct-18 | Sagami bay | 418 | 635 |
| A | Scombridae | 08-Oct-18 | Sagami bay | 370 | 490 |
| A | Scombridae | 08-Oct-18 | Sagami bay | 330 | 392 |
| A | Scombridae | 15-Sep-18 | Sagami bay | 290 | 257 |
| A | Scombridae | 22-Sep-18 | Sagami bay | 272 | 266 |
| A | Scombridae | 22-Sep-18 | Sagami bay | 270 | 249 |
| A | Scombridae | 06-Oct-18 | Sagami bay | 389 | 1003 |
| A | Scombridae | 18-Nov-18 | Sagami bay | 290 | 406 |
| A | Siganidae | 15-Oct-17 | Ishigaki Island | 160 | 130 |
| A | Tetraodontidae | 03-Dec-17 | Tokyo bay | 180 | 196 |
| A | Tetraodontidae | 15-Oct-17 | Ishigaki Island | 100 | 107 |
| A | Tetraodontidae | 16-Dec-17 | Tokyo bay | 150 | 172 |
| A | Tetraodontidae | 16-Dec-17 | Tokyo bay | 130 | 90 |
| A | Tetraodontidae | 16-Dec-17 | Tokyo bay | 130 | 81 |
| A | Tetraodontidae | 13-Jan-18 | Tokyo bay | 204 | 3630 |
| A | Tetraodontidae | 28-Apr-18 | Sakata port | 166 | 130 |
| A | Tetraodontidae | 17-Apr-12 | Tottori port | 155 | 184 |
| A | Tetraodontidae | 17-Apr-12 | Tottori port | 150 | 132 |
| A | Tetraodontidae | 15-Sep-18 | Sagami bay | 239 | 449 |
| A | Tetraodontidae | 15-Sep-18 | Sagami bay | 209 | 311 |
| A | Triglidae | 30-Jul-16 | Sagami bay | 255 | 346 |
| A | Triglidae | 28-Apr-18 | Sakata port | 175 | 139 |
| A | Triglidae | 28-Apr-18 | Sakata port | 170 | 125 |
| A | Triglidae | 25-Oct-15 | Ishigaki Island | 227 | 230 |
| A | Triglidae | 20-Jul-15 | Torinoumi | 225 | 220 |
| A | -^*2^ | 11-Feb-13 | Tokyo bay | 517 | 909 |
| B | Acropomatidae | 06-Feb-16 | Sagami bay | 276 | 517 |
| B | Acropomatidae | 26-Dec-15 | Sagami bay | 210 | 194 |
| B | Branchiostegidae | 06-Oct-18 | Sagami bay | 240 | 406 |
| B | Hexagrammidae | 16-Dec-17 | Tokyo bay | 145 | 71 |
| B | Hexagrammidae | 23-Dec-17 | Horomui port | 210 | 113 |
| B | Hexagrammidae | 23-Dec-17 | Horomui port | 198 | 92 |
| B | Hexagrammidae | 23-Dec-17 | Horomui port | 190 | 101 |
| B | Hexagrammidae | 23-Dec-17 | Horomui port | 200 | 119 |
| B | Hexagrammidae | 23-Dec-17 | Horomui port | 180 | 81 |
| B | -Hexagrammidae | 23-Dec-17 | Horomui port | 190 | 86 |
| B | Hexagrammidae | 23-Dec-17 | Horomui port | 221 | 133 |
| B | Hexagrammidae | 23-Dec-17 | Horomui port | 200 | 95 |
| B | Hexagrammidae | 23-Dec-17 | Horomui port | 205 | 110 |
| B | Hexagrammidae | 23-Dec-17 | Horomui port | 185 | 68 |
| B | Hexagrammidae | 03-Jan-18 | Tokyo bay | 180 | 106 |
| B | Hexagrammidae | 08-Jan-18 | Tokyo bay | 163 | 81 |
| B | Hexagrammidae | 28-Apr-18 | Sakata port | 236 | 250 |
| B | Hexagrammidae | 28-Apr-18 | Sakata port | 240 | 225 |
| B | Hexagrammidae | 28-Apr-18 | Sakata port | 235 | 192 |
| B | Hexagrammidae | 28-Apr-18 | Sakata port | 226 | 252 |
| B | Hexagrammidae | 13-Nov-16 | Tokyo bay | 180 | 109 |
| B | Hexagrammidae | 25-May-18 | Tokyo bay | 155 | 84 |
| B | Hexagrammidae | 06-Dec-12 | Natori river | 280 | 494 |
| B | Hexagrammidae | 06-Dec-12 | Natori river | 170 | 94 |
| B | Hexagrammidae | 10-Aug-13 | Water off Watari | 342 | 883 |
| B | Hexagrammidae | 10-Aug-13 | Water off Watari | 343 | 952 |
| B | Hexagrammidae | 10-Aug-13 | Water off Watari | 242 | 296 |
| B | Hexagrammidae | 08-Dec-12 | Natori river | 240 | 281 |
| B | Hexagrammidae | 07-Dec-12 | Matsuhima bay | 205 | 147 |
| B | Hexagrammidae | 25-Jan-14 | Water off Tashiro | 234 | 234 |
| B | Hexagrammidae | 10-Jan-16 | Tokyo bay | 240 | 304 |
| B | Hexagrammidae | 23-Jan-16 | Tokyo bay | 210 | 178 |
| B | Kyphosidae | 09-Jul-17 | Tokyo bay | 140 | 107 |
| B | Kyphosidae | 01-Jul-17 | Tokyo bay | 290 | 850 |
| B | Kyphosidae | 01-Jul-17 | Tokyo bay | 280 | 792 |
| B | Kyphosidae | 01-Jul-17 | Tokyo bay | 290 | 844 |
| B | Kyphosidae | 01-Jul-17 | Tokyo bay | 280 | 780 |
| B | Kyphosidae | 24-Jun-17 | Tokyo bay | 115 | 58 |
| B | Kyphosidae | 05-Aug-17 | Tokyo bay | 130 | 114 |
| B | Kyphosidae | 05-Aug-17 | Tokyo bay | 115 | 73 |
| B | Kyphosidae | 20-Aug-17 | Tokyo bay | 140 | 128 |
| B | Kyphosidae | 20-Aug-17 | Tokyo bay | 115 | 63 |
| B | Kyphosidae | 15-Jul-17 | Tokyo bay | 165 | 151 |
| B | Kyphosidae | 15-Jul-17 | Tokyo bay | 160 | 133 |
| B | Kyphosidae | 15-Jul-17 | Tokyo bay | 140 | 91 |
| B | Kyphosidae | 15-Jul-17 | Tokyo bay | 120 | 70 |
| B | Kyphosidae | 15-Jul-17 | Tokyo bay | 125 | 60 |
| B | Kyphosidae | 07-Jul-17 | Sagami bay | 218 | 395 |
| B | Kyphosidae | 07-Jul-17 | Sagami bay | 225 | 372 |
| B | Kyphosidae | 07-Jul-17 | Sagami bay | 225 | 406 |
| B | Kyphosidae | 07-Jul-17 | Sagami bay | 220 | 403 |
| B | Kyphosidae | 07-Jul-17 | Sagami bay | 210 | 311 |
| B | Kyphosidae | 07-Jul-17 | Sagami bay | 230 | 441 |
| B | Kyphosidae | 07-Jul-17 | Sagami bay | 220 | 367 |
| B | Kyphosidae | 07-Jul-17 | Sagami bay | 230 | 390 |
| B | Kyphosidae | 07-Jul-17 | Sagami bay | 235 | 406 |
| B | Kyphosidae | 07-Jul-17 | Sagami bay | 240 | 463 |
| B | Kyphosidae | 07-Jul-17 | Sagami bay | 235 | 441 |
| B | Kyphosidae | 07-Jul-17 | Sagami bay | 210 | 341 |
| B | Kyphosidae | 07-Jul-17 | Sagami bay | 240 | 465 |
| B | Kyphosidae | 07-Jul-17 | Sagami bay | 230 | 472 |
| B | Kyphosidae | 07-Jul-17 | Sagami bay | 240 | 473 |
| B | Kyphosidae | 07-Jul-17 | Sagami bay | 215 | 317 |
| B | Kyphosidae | 07-Jul-17 | Sagami bay | 235 | 478 |
| B | Kyphosidae | 07-Jul-17 | Sagami bay | 220 | 347 |
| B | Kyphosidae | 21-Jul-17 | Water off Wakayama | 360 | 1371 |
| B | Kyphosidae | 21-Jul-17 | Water off Wakayama | 340 | 1081 |
| B | Kyphosidae | 21-Jul-17 | Water off Wakayama | 310 | 1077 |
| B | Kyphosidae | 21-Jul-17 | Water off Wakayama | 320 | 1001 |
| B | Kyphosidae | 29-Jul-17 | Tokyo bay | 135 | 115 |
| B | Labridae | 22-Nov-17 | Ishigaki Island | 232 | 107 |
| B | Labridae | 17-Apr-12 | Water off Tottori | 174 | 168 |
| B | Labridae | 17-Apr-12 | Water off Tottori | 154 | 118 |
| B | Labridae | 17-Apr-12 | Water off Tottori | 149 | 108 |
| B | Labridae | 08-Apr-17 | Okinawa Islands | 242 | 485 |
| B | Labridae | 08-Apr-17 | Okinawa Islands | 220 | 343 |
| B | Labridae | 22-Oct-18 | Okinawa Islands | 199 | 272 |
| B | Lutjanidae | 08-Apr-17 | Okinawa Islands | 211 | 374 |
| B | Moridae | 20-Feb-16 | Tokyo bay | 250 | 196 |
| B | Moridae | 20-Feb-16 | Tokyo bay | 205 | 110 |
| B | Moridae | 20-Feb-16 | Tokyo bay | 175 | 71 |
| B | Moridae | 20-Feb-16 | Tokyo bay | 195 | 89 |
| B | Salmonidae | 11-Mar-18 | Lake Ashinoko | -^*1^ | -^*1^ |
| B | Salmonidae | 11-Mar-18 | Lake Ashinoko | 215 | 176 |
| B | Salmonidae | 11-Mar-18 | Lake Ashinoko | 205 | 177 |
| B | Salmonidae | 11-Mar-18 | Lake Ashinoko | 210 | 178 |
| B | Salmonidae | 11-Mar-18 | Lake Ashinoko | 205 | 151 |
| B | Salmonidae | 25-Mar-18 | Lake Ashinoko | 133 | 32 |
| B | Salmonidae | 25-Mar-18 | Lake Ashinoko | 233 | 222 |
| B | Salmonidae | 25-Mar-18 | Lake Ashinoko | 218 | 181 |
| B | Salmonidae | 25-Mar-18 | Lake Ashinoko | 208 | 162 |
| B | Salmonidae | 25-Mar-18 | Lake Ashinoko | 209 | 162 |
| B | Scorpaenidae | 17-Aug-14 | Tokyo bay | 256 | 267 |
| B | Sebastidae | 28-Feb-16 | Sagami bay | 190 | 159 |
| B | Sebastidae | 31-Jul-16 | Water off Toba | 185 | 248 |
| B | Sebastidae | 31-Jul-16 | Water off Toba | 160 | 120 |
| B | Sebastidae | 31-Jul-16 | Water off Toba | 170 | 145 |
| B | Sebastidae | 31-Jul-16 | Water off Toba | 160 | 117 |
| B | Sebastidae | 31-Jul-16 | Water off Toba | 140 | 82 |
| B | Sebastidae | 31-Jul-16 | Water off Toba | 145 | 86 |
| B | Sebastidae | 31-Jul-16 | Water off Toba | 140 | 101 |
| B | Sebastidae | 31-Jul-16 | Water off Toba | 120 | 52 |
| B | Sebastidae | 31-Jul-16 | Water off Toba | 159 | 105 |
| B | Sebastidae | 31-Jul-16 | Water off Toba | 138 | 79 |
| B | Sebastidae | 31-Jul-16 | Water off Toba | 126 | 65 |
| B | Sebastidae | 19-Feb-17 | Sagami bay | 185 | 233 |
| B | Sebastidae | 23-Dec-17 | Otaru port | 125 | 67 |
| B | Sebastidae | 29-Sep-17 | Yoichi port | 190 | 224 |
| B | Sebastidae | 29-Sep-17 | Yoichi port | 170 | 125 |
| B | Sebastidae | 29-Sep-17 | Yoichi port | 135 | 104 |
| B | Sebastidae | 02-Jun-12 | Water off Tottori | 195 | 251 |
| B | Sebastidae | 02-Jun-12 | Water off Tottori | 190 | 224 |
| B | Sebastidae | 02-Jun-12 | Water off Tottori | 177 | 162 |
| B | Sebastidae | 15-Mar-14 | Tokyo bay | 173 | 134 |
| B | Sebastidae | 10-Aug-13 | Water off Watari | 216 | 365 |
| B | Sebastidae | 28-Jun-15 | Tokyo bay | 199 | 240 |
| B | Serranidae | 18-Jun-17 | Okinawa Islands | 260 | 364 |
| B | Serranidae | 28-Jan-17 | Ishigaki Island | 185 | 183 |
| B | Serranidae | 28-Jan-17 | Ishigaki Island | 215 | 370 |
| B | Serranidae | 28-Jan-17 | Ishigaki Island | 185 | 215 |
| B | Serranidae | 28-Jan-17 | Ishigaki Island | 180 | 184 |
| B | Serranidae | 28-Jan-17 | Ishigaki Island | 200 | 201 |
| B | Serranidae | 28-Jan-17 | Ishigaki Island | 185 | 161 |
| B | Serranidae | 08-Apr-17 | Okinawa Islands | 265 | 473 |
| B | Serranidae | 08-Apr-17 | Okinawa Islands | 224 | 425 |
| B | Serranidae | 08-Apr-17 | Okinawa Islands | 200 | 209 |
| B | Serranidae | 08-Apr-17 | Okinawa Islands | 180 | 154 |
| B | Serranidae | 08-Apr-17 | Okinawa Islands | 415 | 1581 |
| B | Serranidae | 08-Apr-17 | Okinawa Islands | 408 | 1501 |
| B | Serranidae | 08-Apr-17 | Okinawa Islands | 223 | 326 |
| B | Serranidae | 28-Jan-17 | Ishigaki Island | 282 | 526 |
| B | Serranidae | 28-Jan-17 | Ishigaki Island | 240 | 379 |
| B | Serranidae | 20-Aug-12 | Water off Hachijojima | 306 | 646 |
| B | Serranidae | 21-Oct-18 | Okinawa Islands | 220 | 255 |
| B | Serranidae | 21-Oct-18 | Okinawa Islands | 214 | 252 |
| B | Serranidae | 06-Oct-18 | Sagami bay | 190 | 196 |
| B | Serranidae | 21-Oct-18 | Okinawa Islands | 250 | 453 |
| B | Sparidae | 12-Nov-16 | Tokyo bay | 155 | 148 |
| B | Synodontidae | 30-Jul-16 | Sagami bay | 330 | 674 |
| B | Synodontidae | 30-Jul-16 | Sagami bay | 330 | 489 |
| B | Synodontidae | 14-Aug-16 | Sagami bay | 275 | 496 |
| B | Synodontidae | 14-Aug-16 | Sagami bay | 210 | 141 |
| B | Synodontidae | 14-Oct-17 | Ishigaki Island | 151 | 62 |
| B | Synodontidae | 26-Aug-18 | Sagami bay | 328 | 605 |
| B | Synodontidae | 26-Aug-18 | Sagami bay | 230 | 213 |

^*1^ No information associated with the fish sample.

^*2^ Species information is unavailable, but the fish is a type of shark belonging to Chondrichthyes.

**Table S2. Classification performance of conventional SVM in terms of the physical properties of various fish muscle samples.**

| **Observed** | **Predicted** | | | | | | **AUC** | | | **Accuracy** | | | **CCR-A** | | | **CCR-B** | | |
| --- | --- | --- | --- | --- | --- | --- | --- | --- | --- | --- | --- | --- | --- | --- | --- | --- | --- | --- |
|  | **Group A** | | | **Group B** | | |  |  |  |  |  |  |  |  |  |  |  |  |
| **Group A** | 89.8 | ± | 3.65 | 99.2 | ± | 3.65 | 0.780 | ± | 0.007 | 0.748 | ± | 0.009 | 0.475 | ± | 0.019 | 0.865 | ± | 0.008 |
| **Group B** | 59.0 | ± | 3.40 | 379 | ± | 3.40 |  |  |  |  |  |  |  |  |  |  |  |  |

**Figure S1. Representative *T*_2_ relaxation curves (a total of 240 curves shown in this figure) for various fish samples used in this study.**


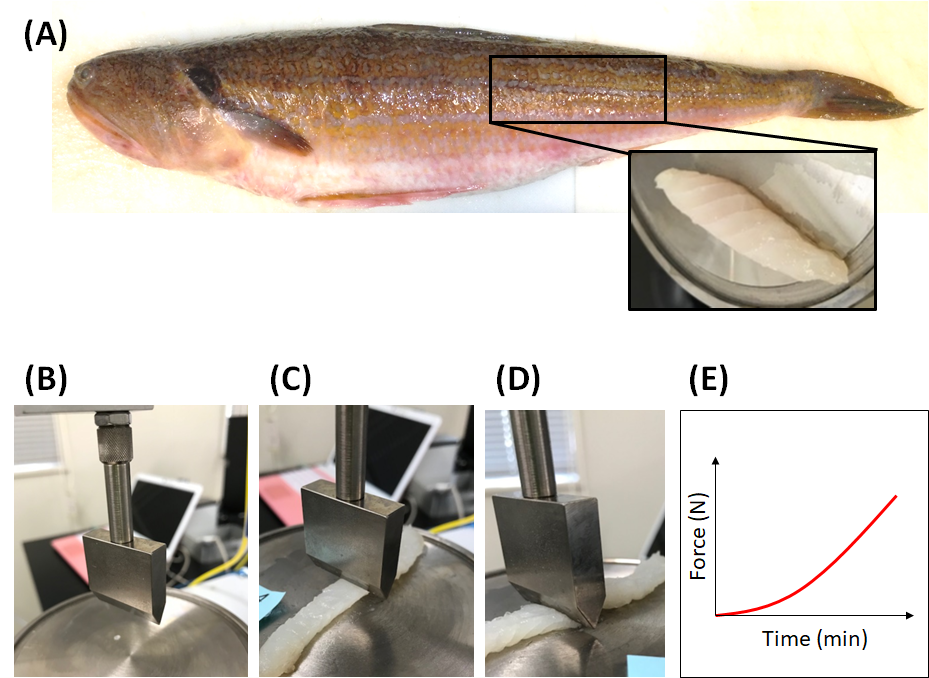


**Figure S2. Analytical procedure involving compressive force measurements by autograph.** Fish muscle above the anal fin was cut into slices (5 mm thick and 10 mm wide), as shown in (A). The fish muscle was placed on an autograph (B, C) and compressed at a constant loading rate of 2 mm min^-1^ over a total distance of 5 mm (D) while recording the force–time curve (E).

**Figure S3. Categorization of compressive force data measured by an autograph using a data-driven approach.** (A) Two-group categorization by HCA. (B) Scatter plot of coefficients *a* and *b* determined by fitting the compressive force data with the exponential function (*y* = *a*e*^b^*^x^). The red and yellow colors indicate fish families belonging to groups A and B, respectively.

**Figure S4. Performance evaluation of the variable optimization approach based on conventional SVM classification** **combined with bootstrap resampling-based matrixing using the hyperparameters of 150 resamples and 100 resampled datasets.** (A) AUC (circles) and accuracy (diamonds). (B) CCR-A (triangles) and CCR-B (squares).

**Figure S5. Comparison of classification performance between SVM-based relaxometric learning (blue bars) and conventional SVM (black bars).** The standard deviations are displayed as error bars.

**Figure S6. Robustness evaluation of the relaxometric learning method for fluctuation of each variable (*T*_2_ relaxation time component).** Mean AUC values in each replaced variable were calculated from the datasets generated by random resampling based on permutation for a variable. The dashed and solid lines indicate original AUC values calculated by conventional ML algorithm (0.78) and the developed relaxometric learning method (0.82), respectively.

**Figure S7. ROC curves and the corresponding AUC values for conventional ML algorithms (dashed lines) and the developed relaxometric learning method (solid lines).** The performance of the RF (purple) and PLS (green) algorithms are shown.

**Figure S8. Classification performance of SVM-based relaxometric learning in determining the geographical differences between Kyphosidae taken from Tokyo Bay and Sagami Bay.** (A) ROC curves and the corresponding AUC values. (B) Comparison of classification performance between SVM-based relaxometric learning (blue bars) and conventional SVM (black bars).
